# Adjuvant alternative cytokine-induced killer cell combined with natural killer cell immunotherapy improves the prognosis of post-mastectomy breast cancer

**DOI:** 10.3389/fimmu.2022.974487

**Published:** 2022-11-10

**Authors:** Xinyi Yang, Desheng Weng, Qiuzhong Pan, Tong Xiang, Chaopin Yang, Zhengrong Wu, Minxing Li, Songzuo Xie, Yan Tang, Jianchuan Xia, Jingjing Zhao

**Affiliations:** ^1^ Collaborative Innovation Center for Cancer Medicine, State Key Laboratory of Oncology in South China, Sun Yat-sen University Cancer Center, Guangzhou, China; ^2^ Department of Biotherapy, Sun Yat-sen University Cancer Center, Guangzhou, China; ^3^ Department of Pathology, School of Basic Medical Sciences, Southern Medical University, Guangzhou, China

**Keywords:** adjuvant cellular immunotherapy, breast cancer, natural killer cells, prognosis, cytokine-induced killer

## Abstract

Breast cancer is one of the most common cancers in women. Triple-negative breast cancer (TNBC) has a significantly worse prognosis due to the lack of endocrine receptors including estrogen receptor (ER), progesterone receptor (PR) and human epidermal growth factor receptor 2 (HER2). In this study, we investigated adjuvant cellular immunotherapy (CIT) in patients with post-mastectomy breast cancer. We enrolled 214 post-mastectomy breast cancer patients, including 107 patients in the control group (who received chemotherapy/radiotherapy/endocrine therapy) and the other 107 patients in the CIT group (who received chemotherapy/radiotherapy/endocrine therapy and subsequent immune cell infusion). Of these 214 patients, 54 had TNBC, including 26 patients in the control group and 28 patients in the CIT group. Survival analysis showed that the overall survival rate of patients treated with cellular immunotherapy was higher than that of patients who were not treated with CIT. Compared to those who received cytokine-induced killer (CIK) cells alone, the patients who received CIK combined with natural killer (NK) cell immunotherapy showed the best overall survival rate. In subgroup analyses, adjuvant CIT significantly improved the overall survival of patients in the TNBC subgroup and the patients who were aged over 50 years. Our study indicates that adjuvant CIK cell combined with NK cell treatment is an effective therapeutic strategy to prolong the survival of post-mastectomy patients, particularly for TNBC patients and those who are aged over 50 years.

## Introduction

Breast cancer is considered to be one of the major diseases that endanger women’s health in the world. On average, one in every 20 women with cancer has breast cancer, and this incidence is as high as one in 8 women in high-income countries (1). The morbidity and the number of patients with breast cancer are also increasing rapidly. In recent years, breast cancer screening projects, such as mammography screening, are included in the physical examination, enabling them to obtain early detection, diagnosis, and treatment. But the mortality of breast cancer is still high, and the number of deaths is also increasing every year.

Triple-negative breast cancer (TNBC) is a breast cancer subtype, which is well known for its high invasiveness and poor prognosis in young women. TNBC accounts for 12% to 17% of all breast cancers. Lack of hormone receptor immunohistochemistry (IHC) staining for estrogen and progesterone without human epidermal growth factor receptor 2 (HER2) protein overexpression or HER2 gene amplification (or both) are the most typical characteristics of TNBC. Due to the lack of receptors, TNBC patients obtain mild benefits from hormone blocking or HER2-specific monoclonal antibody treatment, which often leads to recurrence and metastasis compared to patients with other types of breast cancer who have received systemic adjuvant chemotherapy or neoadjuvant chemotherapy (2). Due to these characteristics, the prognosis of TNBC is poor and there is a lack of effective therapeutic targets (3). Therefore, there is an urgent need for a safer, more applicable, and more effective treatment.

In the 1960s, adoptive cellular immunotherapy (CIT) was first used in tumor therapy. After decades of development, it has made greater achievements. Tumor-reactive T cells (4) and cytokine-induced killer (CIK) cells were infused into patients with cancer to provide antitumor immunity (5).

Natural killer (NK) cells are an important part of lymphocytes, which can directly kill tumor cells and virus-infected cells; thus, they play an important role in immune surveillance and early anti-infection immunity (6). They can mediate spontaneous cytotoxicity and rapidly secrete large amounts of cytokines and chemokines to promote subsequent adaptive immune responses and recruit other lymphocytes (7). The recognition of target cells by NK cells is no limitation of major histocompatibility complex (MHC) molecules. Unlike T cells, NK cells lack the surface T cell receptor and do not cause graft-versus-host disease (GVHD) (8), which makes them a promising strategy for tumor patients.

Purified NK cells have good selective cytotoxicity in osteosarcoma (9), B-cell leukemia, myeloma (10), acute myeloid leukemia (6, 11), lymphoma (12), and some solid tumors, which can reduce tumor recurrence and metastasis. NK cell-based therapies are emerging as safe and efficacious treatments for some cancers (13). The “off-shelf” NK cell therapeutic product oNKord, has received an orphan drug designation from EMA and FDA for treating AML patients, which improved survival in year 1 of 80% *vs*. 35% in the control arm (14).The number and activity of tumor-infiltrating NK cells in colorectal cancer (15) and ovarian cancer (16) are also associated with prognosis. A study shows, in stage III colorectal cancer, the 5-year relapse free survival rate of patients with extensive NK cells infiltration was 80%, was 52% for those with moderate NK cell infiltration, and was 49% for those with little NK cell infiltration. High NK infiltration can improve the prognosis of patients with stage III colorectal cancer (15).This suggests that NK cells may be an independent prognostic factor.

CIK cells are a group of heterogeneous cells, including CD3^+^ CD4^+^ T cell, CD3^+^ CD8^+^ T cell, CD3^-^CD56^+^ NK cell and CD3^+^ CD56^+^ NKT cells, which are cytotoxic against autologous and allogeneic tumors (17, 18). The aim of tumor immunotherapy is to enhance the anti-tumor ability of immune cells, stimulate tumor-specific immunity, reactivate immune cells, and finally achieve the purpose of anti-tumor immunity. CIK cell therapy eliminates the activation of tumor cells and kills them by infusing expanded immune cells (19, 20). In previous studies, CIK cell therapy was considered to play a strong antitumor role in renal cell carcinoma (21), hepatocellular carcinoma(HCC) (5), non-small cell lung cancer (22), and colorectal cancer (23). A retrospective study showed that CIK cells could significantly improve OS of HCC patients (24).Results from another phase III trial showed that CIK cell therapy extended recurrences-free survival to 44 months in patients with HCC (5).

In this retrospective study, we recorded the follow-up survival of breast cancer patients in the experimental group and control group. Our retrospective study showed that the patients received CIK combined with NK cell adoptive immunotherapy had a better prognosis, especially in patients who were aged over 50 years and who were TNBC.

## Materials and methods

### Patient population

This article reviews the medical records of 214 breast cancer patients between July 20, 2005, and September 15, 2012 from a computerized database of the Sun Yat-Sen University Cancer Center (Guangzhou, China). This database recorded the clinicopathological features of patients, including age, tumor size, pathologic grade, TNM (tumor-node-metastasis) stage, hormone receptor (including ER, PR, and HER2), treatment, and outcome. All patients underwent surgery, including quadrantectomy or mastectomy and axillary lymph node dissection. In addition, most patients subsequently received chemotherapy, radiotherapy, and endocrine therapy, which are dependent on their clinical stage. After completing the systemic comprehensive treatment, if there was no organic dysfunction, systemic immunosuppressive treatment, and active autoimmune disease, some patients received at least four cycles of CIT after consent, and no serious adverse events occurred during the CIT.

Patients were excluded from the study based on the following criteria: the presence of a distant metastasis at diagnosis, a history of other malignancies, treatment with adjuvant chemotherapy/radiotherapy, patients who did not receive any chemotherapy/radiotherapy/endocrine therapy after mastectomy and patients who received CIK treatment after recurrence. The decision about inclusion of patients in this retrospective study was made by a multidisciplinary team, which consisted of surgeons, oncologists, physicians, immunologists, and radiologists. After review, 214 patients met the study criteria and were included for further analysis.

Among them, 107 patients had received CIT treatment (CIT group), while the other 107 patients who were diagnosed on the same day or nearly one day but who did not receive CIT were used as controls. Of the 107 patients in the CIT group, 59 received only CIK cell therapy (CIK group) and 48 received alternating CIK and NK cell therapy (CIK + NK group). **Tables 1**, **2** summarize the characteristics of patients in each group.

**Table 1:** Demographics and disease characteristics of patients in the control group patients and the cellular immunotherapy group patients.

Variables	Control group (n = 107)	CIT group (n = 107)	*p* value
**Age (years)**	51.74 ± 11.58	49.00 ± 10.77	0.075
**Tumor Size**			0.336
**≤20**	42	38	
**>20**	65	69	
**Pathologic grade**			0.485
** 1**	11	6	
** 2**	61	64	
** 3**	35	37	
**Tumor stage**			0.717
** T0-1**	39	37	
** T2**	51	60	
** T3**	17	10	
**Node stage**			0.067
** N0**	19	16	
** N1**	50	41	
** N2**	34	37	
** N3**	4	13	
**TNM stage**			0.340
** I**	15	11	
** II**	43	41	
** III**	49	55	
**Hormone receptor**			
**ER**			1.000
** Positive**	44	45	
** Negative**	63	62	
**PR**			1.000
** Positive**	52	51	
** Negative**	55	56	
**HER2**			0.407
** Positive**	58	65	
** Negative**	49	42	
**TNBC**			0.088
** Yes**	26	28	
** No**	81	79	
**Therapy**			
**Chemotherapy**			1.000
** Yes**	97	96	
** No**	10	11	
**Radiotherapy**			1.000
** Yes**	98	98	
** No**	9	9	
**Endocrine therapy**			0.779
** Yes**	67	64	
** No**	40	43	

CIT, cellular immunotherapy; TNM, tumor-node-metastasis; ER, estrogen; PR, progesterone; HER2, human epidermal growth factor receptor 2; TNBC, triple-negative breast cancer.

**Table 2:** Demographics and disease characteristics of patients in the CIK immunotherapy treatment group and CIK plus NK cells immunotherapy treatment group.

Variables	CIK group (n = 59)	CIK+NK group (n = 48)	*p* value
**Age (years)**	48.61 ± 11.07	49.48 ± 10.48	0.680
**Tumor Size**			0.150
** ≤20**	24	14	
** >20**	35	34	
**Pathologic grade**			0.051
** 1**	2	4	
** 2**	35	29	
** 3**	26	11	
**Tumor stage**			0.107
** T0-1**	24	13	
** T2**	31	29	
** T3**	4	6	
**Node stage**			0.984
** N0**	8	8	
** N1**	24	17	
** N2**	20	17	
** N3**	7	6	
**TNM stage**			0.802
** I**	5	6	
** II**	25	16	
** III**	29	26	
**Hormone receptor**			
**ER**			0.696
** Positive**	26	19	
** Negative**	33	29	
**PR**			0.173
** Positive**	32	19	
** Negative**	27	29	
**HER2**			1.000
** Positive**	36	29	
** Negative**	23	19	
**TNBC**			0.659
** Yes**	14	14	
** No**	45	34	
**Therapy**			
**Chemotherapy**			1.000
** Yes**	53	43	
** No**	6	5	
**Radiotherapy**			1.000
** Yes**	54	44	
** No**	5	4	
**Endocrine therapy**			0.555
** Yes**	37	27	
** No**	22	21	

CIK, cytokine-induced killer; NK, natural killer; TNM, tumor-node-metastasis; ER, estrogen; PR, progesterone; HER2, human epidermal growth factor receptor 2; TNBC, triple-negative breast cancer.

### Treatment procedures

After completing sequential radiotherapy/chemotherapy over two weeks and when the blood routine examination result had returned to normal, 50-60 ml of heparinized peripheral blood of patients was obtained for cell culture. The blood routine examination result return to normal was defined as white blood cell count≥3.0×10^9^/L, absolute neutrophil count≥1.5×10^9^/L, platelet count≥75×10^9^/L, hemoglobin≥9g/dL, and absolute lymphocyte count≥0.7×10^9^/L. Before the first infusion of cells, the blood was obtained again for the next cycle of cellular therapy. After the first blood sampling and standardized culture for two weeks, the cells after detection were re-infused into the patients. Heparinized peripheral blood was obtained for the next cell culture before each time of cell infusion.

For the CIK group, blood was collected at week 0 and CIK cells was performed at week 2. The peripheral blood needed by week 4 was collected before infusion at week 2.

**Figure 1:**
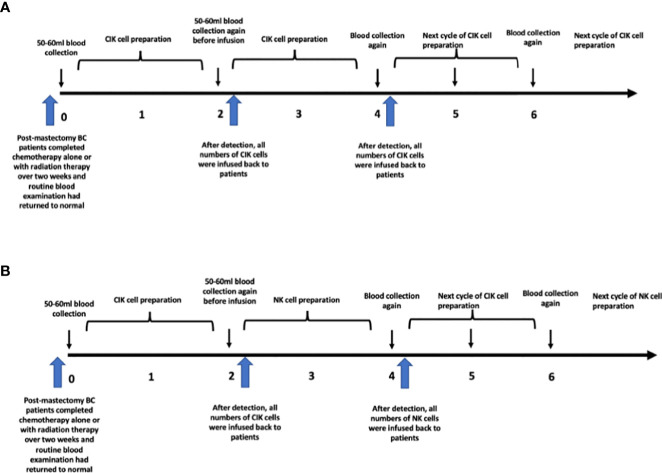
Cells cultured process in different groups. **(A)** CIK group. **(B)** CIK+NK group.

However, for the CIK + NK group, the cell culture was slightly different. Blood was obtained for CIK cell culture at week 0, and CIK cells were infused at week 2. Blood was collected again for expanded NK cell culture before infusion at week 2. From week 2 to week 4, peripheral blood cells were cultured to induce differentiation into expanded NK cells, and they were finally infused at week 4. After that, CIK cells were infused again at week 6, and expanded NK cells were infused at week 8. In short, CIK and expanded NK cells were treated alternately at an interval of two weeks. The details of the treatment procedure are shown in **Figure 1**.

### Generation of immune cells

The preparation methods of CIK and NK have been introduced in our previous studies (25). In short, 50-60 ml heparinized peripheral blood was obtained 2 weeks after the patients completed radiotherapy, chemotherapy, and endocrine therapy, and the blood routine examination results returned to normal. Peripheral blood mononuclear cells (PBMCs) were isolated by Ficoll-Hypaque gradient centrifugation and cultured in X-VIVO (Lonza) serum-free medium for 24 hours, supplemented with 1,000 U/mL recombinant human interferon-gamma (IFN-γ) (Shanghai Clone Company) at 2 x 10^6^ cells/ml and incubated at 37°C in a humidified atmosphere containing 5% CO_2_ for 24h. Then, the following were added: 100 ng/ml mouse anti human CD3 monoclonal antibody (R & D Systems, Shanghai, China), 100 U/ml recombinant human interleukin-1α (Life Technologies, Guangzhou, China), and 1000 U/ml recombinant human interleukin-2 (rhIL-2; Beijing Sihuan, Beijing, China). Fresh medium and fresh rhIL-2 were added every 2 days and cell density was maintained at 2 x 10^6^ cells/ml.

To amplify NK cells, PBMCs were isolated by Ficoll-Hypaque gradient centrifugation and cultured in X-VIVO (Lonza) serum-free medium in a T75 culture flask coated with HER2 monoclonal antibody(Shanghai Roche, Shanghai, China)at 1.5 x 10^6^ cells/mL and incubated at 37°C in a humidified atmosphere containing 5% CO_2_ for 24h. After that, the following were added: 1000 U/ml rhIL-2 (Beijing Sihuan, Beijing, China), 25ug/ml recombinant human interleukin-15(PeproTech, USA). After 4 days, the cells were centrifuged, and the supernatant was discarded and transferred to the X-VIVO serum-free medium containing 1000 U/ml rhIL-2 for 2 weeks. Fresh medium and fresh rhIL-2 were added every 2 days and cell density was maintained at 1.5 x 10^6^ cells/ml.

### Phenotypic analysis of CIK and NK cells

**Figure 2:**
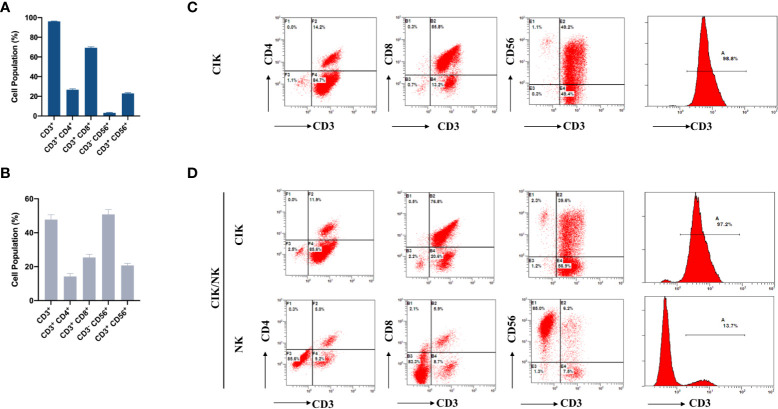
Phenotypic analysis of CIK cells and NK cells in breast cancer patients after the first expansion. **(A)** The phenotype of autologous CIK cells from 65 patients in each cycle was evaluated using flow cytometry. The percentage of CD3^+^, CD3^+^ CD4^+^, CD3^+^ CD8^+^, CD3-CD56^+^ and CD3^+^ CD56^+^ are shown. **(B)** The phenotype of autologous NK cells from 24 patients in each cycle was evaluated using flow cytometry. The percentage of CD3^+^, CD3^+^ CD4^+^, CD3^+^ CD8^+^, CD3-CD56^+^ and CD3^+^ CD56^+^ are shown. **(C)** Typical flow cytometry analysis of CD3^+^CD4^+^T cells, CD3^+^CD8^+^T cells, CD3^+^CD56^+^NKT cells, and CD3−CD56^+^NK cells after expansion in CIK group. **(D)** Typical flow cytometry analysis was performed on two different types of expanded cells from CIK/NK patients. The data were collected from the same patient. The results were from 89 CIT patients and are represented as mean ± SEM.

To meet the different needs, CIK and NK cells were obtained according to different cell expansion and culture methods. The survival rate of the two cell types was more than 95%. For the CIK phenotype, the final number of immune cells produced was approximately 1.0×10^10^-1.2×10^10^. The median percentages of CD3^+^, CD3^+^ CD4^+^, CD3^+^ CD8^+^, CD3^-^ CD56^+,^ and CD3^+^ CD56^+^ populations in the CIK group in final immune cells were 97.75% (range, 43.1-99.5%), 24.3% (range, 1.7-74.3%), 70.35% (range, 17-93.3%), 1.6% (range, 0.2-55.7%), and 21.2% (range, 4.3-66.3%), respectively (**Figure 2A**).

Compared to the CIK phenotype, the final number of immune cells was 4.2×10^9^-5.8×10^9^, while the median percentages of CD3^+^, CD3^+^ CD4^+^, CD3^+^ CD8^+^, CD3^-^ CD56^+^, and CD3^+^ CD56^+^ in the NK group were 45.25% (range, 9.3-97.5%), 8.3% (range, 1-81.6%), 19.05 (range, 2.7-71%), 53.5% (range, 1.5-89.9%), and 18.45% (range, 4.6-67.0%), respectively (**Figure 2B**).

### Follow-up

After surgery, all patients were postoperative followed-up included clinical and phone-call inquiring every 2 months in the first year at our outpatient department or follow-up center, every 3 months in the second year, every 6 months from the 3rd to 5th years, and annually thereafter at least until 5 years after the operation or until the patient died, whichever came first. In this study, the last follow-up was December 30, 2021. The telephonic follow-up included the following: enquiring about the general physical condition of patients and asking whether there was any recurrence and metastasis of tumors and providing corresponding medical suggestions. The patients’ blood routine examination, biochemical routine examination, tumor markers, computed tomography, bone density determination, bone scintigraphy, and B-ultrasound of bilateral axillary and cervical lymph nodes were monitored at each re-examination.

Overall survival (OS) was defined according to the National Cancer Institute’s Response Evaluation Criteria in Solid Tumors (26). OS was calculated from the date of definitive surgery to death resulting from any cause or was censored at the last follow-up. If recurrence or metastasis was confirmed during follow-up, our multidisciplinary team provided other medical suggestions, including surgery, chemotherapy besides the anthracycline- and taxane-based regimen, or radiation treatment. For those patients whose endocrine receptors or HER2 were positive, appropriate and regular endocrine therapy was needed. Supportive treatment was given to patients who were intolerant to any systemic and local treatment. All toxicities were graded according to the National Cancer Institute Common Toxicity Criteria for Adverse Events version 4.0.

### Statistical analysis

Pearson X^2^ and Fisher exact tests were used to compare categorical variables. Two-way ANOVA was used to explore the differences in cell phenotypes between four different cycles. OS curves were constructed according to the Kaplan-Meier method and compared using the log rank test. OS of patients was defined as the interval from completion of surgery to death or the last follow-up. Multivariate analysis was performed after univariate analysis found statistically significant variables. Hazard ratio and the 95% confidence interval were also provided.

In all analyses, a difference of 0.05 was considered significant. All statistical evaluations were performed using SPSS software (version 26.0 of IBM’s Social Science Statistical Software Package) and GraphPad Prism 9 (version 9 of GraphPad software company).

## Results

### Patient demographics and clinical characteristics

A total of 214 patients with breast cancer were retrospectively included in the study. Among them, 107 patients received traditional breast cancer treatment without CIT, while the other 107 patients received traditional treatment and sequential CIT. The baseline characteristics of patients are shown in **Tables 1**, **2**. There were no statistically significant differences in the demographic or clinical characteristics between the two groups. In this study, 54 patients had TNBC, the other 160 patients had non-TNBC. The median age was 50.29 years (range, 30 to 82 years).

### Phenotypic analysis of final immune cells

To determine whether the phenotype of expanded cells in the CIK group and NK group had changed, we analyzed the difference between the two phenotypes. Compared with the CIK group, CD3^-^ CD56^+^ were significantly increased, while CD3^+^, CD3^+^ CD4^+^, CD3^+^ CD8^+^ and CD3^+^CD56^+^ were decreased in the NK group (**Figures 2A, B**). Besides, we collected immune cells from CIK group and CIK/NK group after different amplification methods for flow cytometric analysis. The positive rate of CD3 in the expanded immune cells was high in CIK group, accounted for 98.8% (**Figure 2C**). Patients in the CIK/NK group were also received highly positive for CD3 immune cell therapy when treated with CIK cells. However, when treated with alternate expanded NK cells, CD3^+^ accounted for only a small population about 13.7%, and CD3^-^ CD56^+^ NK cells accounted for 85% of the reinfused immune cells (**Figure 2D**). Flow cytometry analysis of different groups in **Figure 2D** is derived from alternating CIK and NK cells expansion of immune cells from the same patient.

Further, to identify whether there was any phenotypic evolution of patients with breast cancer after CIK cell only infusion or CIK and NK cell combined infusion. The phenotypes of 63 patients in the CIK and CIK+NK groups from the first cycle to the fourth cycle were included in the analysis. After 14 days of amplification, the number of cells with different phenotypes in different cycles and different groups were analyzed.

**Figure 3:**
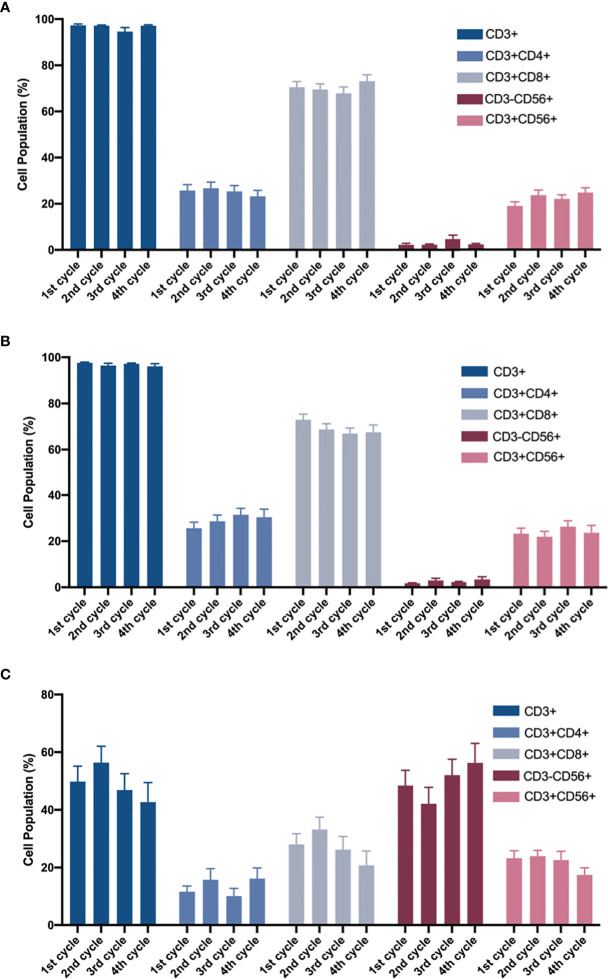
After 14-day of amplification, the proportions of different cell populations in the four cycles. **(A)** CIK group and **(B, C)** CIK + NK group were analyzed by two-way ANOVA. The cell population of the phenotypes in **(A)** CIK group, CIK + NK group during **(B)** CIK culture and **(C)** NK culture has been shown. For the same cell population, neither CIK group nor CIK+NK group, was no statistical difference between any two cycles. The results are represented as mean ± SEM.

In the expansion of CIK cells, neither the CIK group nor the CIK+NK group showed a significant difference among CD3^+^, CD3^+^ CD4^+^, CD3^+^ CD8^+^, CD3^-^CD56^+^, and CD3^+^ CD56^+^ (**Figures 3A, B**). Even in the NK group, no statistically significant differences were found between CD3^+^, CD3^+^ CD4^+^, CD3^+^ CD8^+^, CD3^-^CD56^+^, and CD3^+^ CD56^+^ cells in the four cycles (**Figure 3C**). Slightly different phenotypes of cells infused in different cycles also indicated that irrespective of whether the patients received CIK cell only infusion or CIK and NK cell combined infusion, they received stable CIT in each cycle.

### Safety and toxicity of adjuvant cell immunotherapy

Overall, the patients treated with adjuvant cell therapy showed good tolerance. No significant toxicity was observed.

Among all patients who received CIT, only 4 patients had self-limiting fever during infusion, 3 patients had palpitations, 7 patients experienced fatigue, 3 patients had arthralgia, 2 patients had nausea and vomiting during treatment, and 3 patients had transient hypertension. CIT-related adverse events are shown in **Table 3**. All the above adverse events were rated as grade 1 or 2 and some of the patients were relieved by symptomatic treatment. No treatment-related serious adverse events, such as pneumonitis, colitis, hepatitis, and treatment-related deaths, were noted in any of the patients.

**Table 3:** CIT related adverse events according to category and grade.

Category	Patient no.		
	Total	CIK group (n = 59)	CIK+NK group (n = 48)
**Fever**	4	2	2
**Palpitation**	3	1	2
**Fatigue**	7	4	3
**Rash**	NA	NA	NA
**Pruritus**	NA	NA	NA
**Arthralgia**	3	2	1
**Anorexia**	NA	NA	NA
**Nausea/vomiting**	2	1	1
**Allergic reaction**	NA	NA	NA
**Hypertension**	3	1	2
**Pneumonitis**	NA	NA	NA
**Hepatitis**	NA	NA	NA
**Colitis**	NA	NA	NA

CIK, cytokine-induced killer; NK, natural killer; NA, not applicable.

### Survival analysis

**Figure 4:**
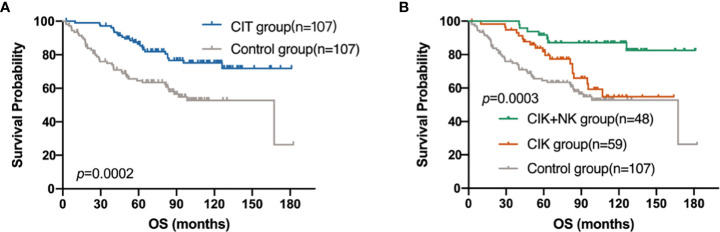
Kaplan–Meier estimates of overall survival (OS) of patients with postoperative breast cancer by different treatment groups. **(A)** OS curve of patients in CIT group (n = 107) versus the control group (n = 107). **(B)** OS curve of patients in CIK+NK group (n = 48), CIK group (n = 59) and the control group (n = 107).

In these 214 patients, the efficacy of CIT was better than that in the control group (*p*=0.0002) (**Figure 4A**). Besides, the CIK combined with NK cell therapy achieved the best prognosis (*p*=0.0003) (**Figure 4B**). Compared with the 1-year, 3-year, 5-year and 10-year survival rates of 98.28%, 94.83%, 81.88% and 54.77% in the CIK group and those of 93.42%, 74.93%, 64.57% and 52.82% in the control group, the 1-year and 3-year OS rate in the CIK+NK group was 100%, 100%, 91.67 and 87.14%, which showed a strong anti-tumor effect and ability to resist tumor recurrence and metastasis for a long time.

### Univariate and multivariate analyses

To determine the prognosis of postoperative breast cancer patients, the variables were further assessed in univariate and multivariate Cox proportional hazards regression analyses. Younger age (*p*=0.044) and receipt of adjuvant CIT (*p*=0.003) showed a significant association with improved OS in both univariate and multivariate analyses (**Table 4**).

**Table 4:** Univariate and multivariate analysis of overall survival for breast patients in the CIT and control groups.

Variable	Univariate analysis			Multivariate analysis	
	HR (95%CI)	*P* value		HR (95%CI)	*P* value
Age (≥50 *vs*.<50)	1.657 (1.014—2.708)	0.044*		1.900 (1.115—3.240)	0.018*
Treatment (CIT *vs*. control)	0.472 (0.290–0.771)	0.003*		0.400 (0.197—0.812)	0.011*
Tumor size (>20 *vs*. ≤20)	1.415 (0.996–2.021)	0.056			
Clinical stage (1 *vs*. 2 3)	1.214 (0.581—2.537)	0.606			
TNBC (yes *vs*. no)	0.654 (0.357–1.196)	0.168			
Pathologic grade (1 *vs*. 2 3)	1.365 (0.858–2.171)	0.190			
ER (pos *vs*. neg)	1.474 (0.911–2.385)	0.114			
PR (pos *vs*. neg)	1.308 (0.813–2.105)	0.268			
Her2 (pos *vs*. neg)	1.056 (0.657–1.698)	0.822			

HR, Hazard ratio; CI, confidence interval; CIT, cellular immunotherapy; TNBC, triple-negative breast cancer; ER, estrogen; PR, progesterone; HER2; human epidermal growth factor receptor 2. *Statistically significant, p < 0.05.

To further explore the variation affecting the different prognoses of patients in the CIT group, univariate and multivariate Cox regression analyses of OS for patients in the CIK and CIK+NK groups were performed (**Table 5**).

**Table 5:** Univariate and multivariate analysis of overall survival for breast patients in the CIK and CIK+NK groups.

Variable	Univariate analysis		Multivariate analysis	
	HR (95%CI)	*P* value	HR (95%CI)	*P* value
**Age (≥50 *vs*.<50)**	1.056 (0.489–2.280)	0.890		
**Treatment (CIK *vs*. CIK+NK)**	3.244 (1.343–7.833)	0.009*	3.244 (1.343–7.833)	0.009*
**Tumor size (≤20 *vs*. >20)**	1.193 (0.518–2.749)	0.678		
**Clinical stage (1 *vs*. 2 3)**	1.326 (0.313–5.613)	0.702		
**TNBC (yes *vs*. no)**	0.328 (0.098–1.092)	0.069		
**Pathologic grade (1 *vs*. 2 3)**	0.769 (0.352–1.676)	0.508		
**ER (pos *vs*. neg)**	1.563 (0.723–3.376)	0.256		
**PR (pos *vs*. neg)**	2.124 (0.958–4.710)	0.064		
**Her2 (pos *vs*. neg)**	1.756 (0.738–4.177)	0.203		

HR, Hazard ratio; CI, confidence interval; CIK, cytokine-induced killer; NK, natural killer; TNBC, triple-negative breast cancer; ER, estrogen; PR, progesterone; HER2, human epidermal growth factor receptor 2. *Statistically significant, p < 0.05.

Irrespective of univariate analysis or multivariate analysis, CIT treatment was associated with better OS as an independent prognostic factor for patients. Moreover, we found that patients with cell reinfusion by alternating CIK and NK cell therapy could achieve greater benefits than patients with CIK therapy only(*p*=0.009).

### Subgroup analysis

**Figure 5:**
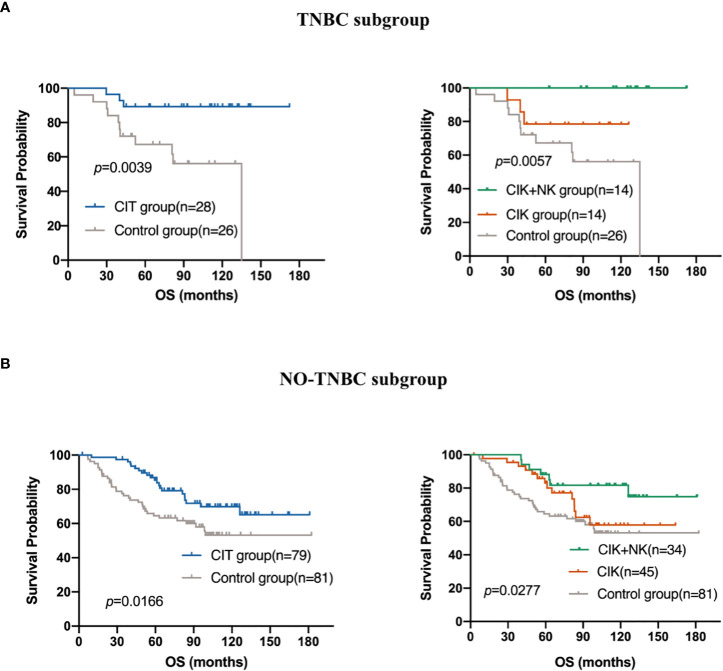
Subgroup analysis to estimate the survival benefits from CIT in **(A)** Triple-negative breast cancer (TNBC)(n=54) and **(B)** NO-TNBC subgroup(n=160).

TNBC is one of the subtypes of breast cancer, and it is characterized by a lack of ER, PR, and HER2 receptors, which makes endocrine therapy after traditional treatment ineffective. Besides, TNBC often indicates poorer prognosis and shorter survival time, and it is more associated with recurrence and metastasis. CIT therapy exerts a wide range of antitumor effects independent of endocrine hormone receptors. Then, we explored whether there was a difference in the prognosis of patients with CIT in the TNBC population. Among the 214 patients, 54 had TNBC, accounting for about a quarter of patients. **Figure 5A** shows that CIT treatment resulted in a better prognosis for patients with TNBC (*p*=0.0039). The effect of alternating treatment with CIK and NK cells was the best (*p*=0.0057). Though CIT significantly improve the patients’ OS in both TNBC subgroup and NO-TNBC group (**Figure 5B**), the patients in TNBC group seem to gain more benefit from adjuvant CIT than NO-TNBC group from CIT. Compared with the 5-year and 10-year survival rates of 89.29% and 89.29% in the TNBC group received CIT treatment, the 5-year and 10-year survival rates in the NO-TNBC group was 85.4% and 65.54%.The difference of 5-year and 10-year survival proportions between CIT group and control group was 19.17% and 25.01% in TNBC group and 15.34% and 4.52% in NO-TNBC group.

To date, according to the follow-up database, all TNBC patients treated with CIK + NK cell therapy are still alive, which shows the great potential and advantages of CIK cell combined with NK cell immunotherapy.

**Figure 6:**
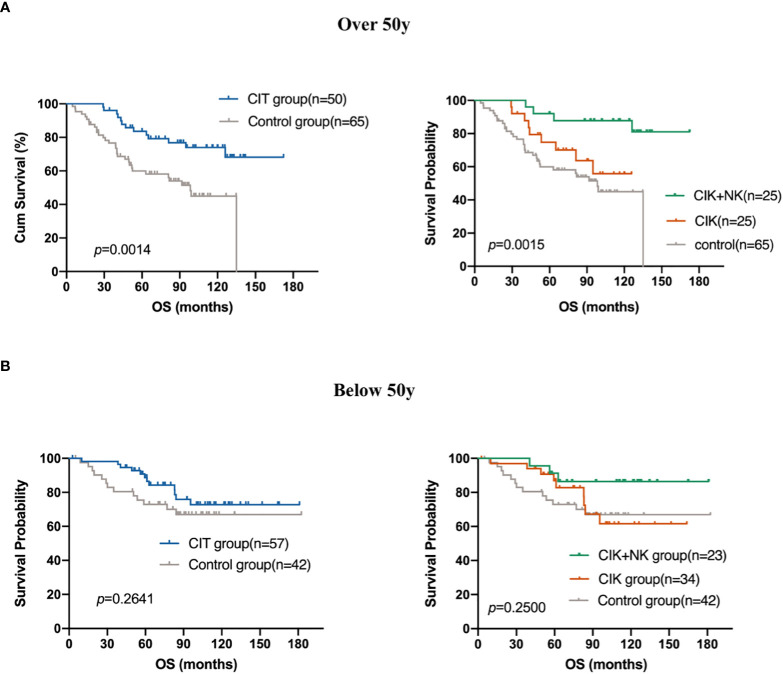
Subgroup analysis to estimate the survival benefits from CIT in patients who are **(A)** over 50 years old (n=115) and who are **(B)** below 50 years old (n=99).

Both univariate and multivariate Cox regression analyses included age (*p* =0.044) into the prognostic equation, and they indicated that the patient’s age was an independent prognostic factor. Patients older than 50 years showed a worse prognosis. This is not very difficult to understand as the immune surveillance, immune killing, and anti-tumor ability of the elderly are not so strong compared with those in the young subjects. Thus, we further explore whether the use of CIT treatment caused a difference in the OS of patients older than 50 years. Survival analysis showed that CIT treatment was a prognostic factor in patients aged over 50 years. The results showed that patients older than 50 years were able to obtain more benefit from CIT treatment comparing with the patients younger than 50 years (*p*=0.0014) (**Figures 6A, B**).

## Discussion

Cellular immunotherapy, such as chimeric antigen receptor-T cell, has been widely applied in hematological tumors (27), and its effectiveness and safety have been widely recognized. However, research on the effect of CIT on solid tumors is still very limited. A few studies have assessed the efficacy and prognosis of CIT in patients with TNBC.

One study showed that the combination of CIK cells and cetuximab restrains the growth of patient-derived TNBC xenografts. Besides, CIK cells and CTX combination acts as an excellent adjuvant therapy to limit metastatic spread after the removal of the primary tumor (28).Two observational studies with small sample sizes of 23 (NCT01395056) and 46 (NCT01232062) patients can be found on *ClincalTrials.gov*. But our study enrolled 214 candidates. The result suggested that patients who received adjuvant CIT achieved longer survival, and patients who received alternate CIK and NK cell treatment exhibited a better prognosis than those who received CIK cells only. Patients with TNBC or those who were aged over 50 years, which may indicate a worse prognosis, can also obtain more benefits from adjuvant CIT.

The cytotoxicity of NK cells is not limited by the MHC. The aim of adjuvant therapy after surgical resection is to eliminate residual tumor cells, reduce the circulating tumor cell load in the blood circulation system as much as possible, and prevent subsequent tumor recurrence and metastasis. Alternative CIK cells and NK cells are directly reinfused into the blood circulation. They are originally components and effector cells of the normal human immune system; hence, they have better selectivity for tumor cells than other types of chemotherapy or radiotherapy, which cannot distinguish between “self” and “non-self”, and normal and abnormal tissue. The aim of adjuvant CIT is to expand the number of immune cells in the peripheral blood of patients. NK cells only account for a small proportion of lymphocytes; about 2% to 18% of peripheral blood (29). The cocktail, amplified *in vitro*, injected after amplification has more killer cells (effector cells) than those in the patients’ original blood circulation, which can kill or maintain the circulating tumor cells that may colonize and metastasize at a low level.

In subgroup analysis, we chose to explore whether there were differences in the use of CIT in TNBC. The results showed that in 54 TNBC patients, the prognosis of patients treated with CIT was better. CIT was also defined as one of the factors that can independently affect the prognosis of TNBC patients. According to our follow-up, 14 patients in the CIT group treated with CIK + NK cells for TNBC have still alive.

Both univariate and multivariate Cox regression analyses included age into the equation; thus, suggesting that age is related to the prognosis of patients. The prognosis of patients older than 50 years was worse than that of patients aged below 50 years. The patients who were aged over 50 years gained more benefit from CIT (*p*=0.0014). Some older patients are limited by several basic diseases or poor physical condition, and they are unable to tolerate surgical treatment and cytotoxicity of whole chemotherapy. If they can achieve longer OS from CIT, it is suggested that CIT may be a reliable treatment for those patients who cannot tolerate any other treatments.

It has been mentioned that CIT after chemotherapy or surgery can prolong the survival time of patients (30), and this treatment is different from the targeted drugs in chemotherapy, which can lead to drug resistance with the increase in drug usage time. Therefore, CIT may be an effective and safe therapy for patients.

However, the current CIT treatment also encounters many problems, such as how to expand adequate number of cells in a short time *in vitro*, how to genetically modify NK cells, and how to obtain a stable source of NK cells. The peripheral blood of patients was collected and stimulated with cytokines to obtain expanded NK cells. Due to the influence of the patient, the proportion of expanded NK cells in the total number of cells was also different. Clinically, the cost of expanding NK cells is quite high. We are trying to find a more economical way to obtain a large number of proliferating NK cells.

The sources of NK cells are peripheral blood, umbilical cord blood, postpartum placenta, and induced pluripotent stem cells (iPSCs) (31). The peripheral blood of patients is most commonly used in the clinic. Peripheral blood was extracted and amplified *in vitro* and then reinfused into the patients. NK cells do not cause GVHD; thus, homologous NK cell reinfusion using normal peripheral blood from patients’ families is also a potential resource, especially for those who are unable to tolerate blood collection or patients whose blood routine examination results do not return to normal before infusion.

## Conclusion

In this retrospective study, we found that the CIT can be a promising remedy for patients with post-mastectomy breast cancer to prolong their OS. Importantly, our findings provided evidence that sequential CIT with alternate application of CIK and NK cells after surgery, chemotherapy, and endocrine therapy causes a better survival improvement, particularly in TNBC patients and patients older than 50 years.

## Data availability statement

The raw data supporting the conclusions of this article will be made available by the authors, without undue reservation.

## Ethics statement

The studies involving human participants were reviewed and approved by Sun-Yat Sen University Cancer Center B2016-036-02. The patients/participants provided their written informed consent to participate in this study.

## Author contributions

JZ and JX contributed to the conception and design. XY, DW, ML and YT conducted the experiments. QP, SX, ZW, CY and TX analyzed and interpreted the data. XY and JZ drafted the manuscript. All authors contributed to the article and approved the submitted version.

## Funding

This work was primarily supported by a grant from the National Natural Science Foundation of China (No. 81402560), the Guangdong Natural Science Foundation (No. 2021A1515010443;2018A0303130344), the Guangdong Province Science and Technology Plan Project (No. 2017A020215029), and Guangdong Esophageal Cancer Institute Science and Technology Program (No. Q201802).

## Conflict of interest

The authors declare that the research was conducted in the absence of any commercial or financial relationships that could be construed as a potential conflict of interest.

## Publisher’s note

All claims expressed in this article are solely those of the authors and do not necessarily represent those of their affiliated organizations, or those of the publisher, the editors and the reviewers. Any product that may be evaluated in this article, or claim that may be made by its manufacturer, is not guaranteed or endorsed by the publisher.
